# Complete Genome Sequence of Klebsiella pneumoniae Siphophage Sugarland

**DOI:** 10.1128/MRA.01014-18

**Published:** 2018-11-15

**Authors:** Samuel G. Erickson, Lauren Lessor, Chandler J. O'Leary, Jason J. Gill, Mei Liu

**Affiliations:** a Center for Phage Technology, Texas A&M University, College Station, Texas, USA; University of California, Riverside

## Abstract

Klebsiella pneumoniae is a Gram-negative bacterium associated with the gastrointestinal tract and is a significant nosocomial pathogen due to its antibiotic resistance. Phage therapy against K. pneumoniae may prove useful in treating infections caused by this bacterium. This announcement describes the genome of the T5-like K. pneumoniae siphophage Sugarland.

## Abstract (precis)

Klebsiella pneumoniae is a Gram-negative bacterium associated with the gastrointestinal tract and is a significant nosocomial pathogen due to its antibiotic resistance. Phage therapy against K. pneumoniae may prove useful in treating infections caused by this bacterium.

## ANNOUNCEMENT

Klebsiella pneumoniae is a Gram-negative bacterium found in soil and the mucosal lining of the intestinal tract. It can cause pneumonia, urinary tract infections, sepsis, and soft tissue infections and is a significant nosocomial pathogen due to its resistance to antibiotics (1, 2). Carbapenemase-producing strains of sequence type 258 (ST258) are among the most prevalent in U.S. clinical centers (3). K. pneumoniae phage Sugarland was isolated from a wastewater treatment plant in College Station, Texas, in October 2016 using a carbapenem-resistant K. pneumoniae ST258 clinical isolate as the host. Upon isolation, it was identified as a siphophage using negative-stain transmission electron microscopy performed at the Texas A&M University Microscopy and Imaging Center.

Phage genomic DNA was prepared as described previously and sequenced on the Illumina MiSeq platform as paired-end 250-bp reads (4). FastQC (https://www.bioinformatics.babraham.ac.uk/projects/fastqc/) was used to quality control reads, and reads were trimmed with the FastX Toolkit (hannonlab.cshl.edu) before being assembled to a single contig at 103.3-fold coverage using SPAdes 3.5.0 (5). Contig completion was confirmed by PCR and sequencing of the resulting product. Along with manual correction, Glimmer3 (6) and MetaGeneAnnotator (7) were used to predict protein-coding genes; tRNA genes were predicted with ARAGORN (8). Sequence similarity searches by BLASTp (9) and conserved domain searches with InterProScan 5 (10) were used to predict protein functions. All analyses were conducted via the CPT Galaxy (11) and WebApollo (12) interfaces (cpt.tamu.edu) using default parameters.

The 111,103-bp double-stranded DNA genome of phage Sugarland has a coding density of 87% and a GC content of 45%, which is significantly lower than the 58% GC content of the host (13). Analysis showed 174 predicted protein-coding genes and 24 identified tRNA genes. The progressiveMauve algorithm (14) was used to compare Sugarland’s nucleotide similarity against the NR database, and the most similar organism at 78% sequence identity was the Klebsiella phage vB_Kpn_IME260 (GenBank accession no. KX845404). BLASTp analysis of the Sugarland proteome showed close homology to other T5-like phages, including the canonical phage T5 itself, with 110 similar proteins (E value < 0.001). Analysis by PhageTerm (15) was unable to precisely determine the extent of the terminal repeats typically associated with T5-like phages, and this genome was reopened to be syntenic to T5 with the predicted *dmp* as the first gene of the genome.

The genome displayed a 1,587-bp noncoding region characteristic of T5-like phages (16). The structural tail fiber and side tail fiber genes were identified, including the tail tip, baseplate, major tail subunit, and L-shaped side tail fiber proteins. Similar to T5, the tape measure chaperone protein of Sugarland did not contain a predicted frameshift signal (17). Genes involved in lysis, including a holin, a d-alanyl-d-alanine carboxypeptidase endolysin, and a partially embedded two-component spanin complex, were identified (18). There were 3 HNH endonucleases identified in the Sugarland genome, but all had free-standing open reading frames (ORFs) and were not introns (19). Interestingly, an NAD^+^-dependent protein deacetylase in the sirtuin-2 family was found. Present in many organisms, protein acetylation helps regulate protein-protein interaction, DNA-protein interaction, and protein stability (20).

### Data availability.

The genome sequence of phage Sugarland was deposited under GenBank accession no. MG459987 and BioSample accession no. SAMN10128164. The BioProject accession number is PRJNA222858, and the SRA accession number is SRR7902581.
